# Molecular approaches to identify and differentiate *Bacillus anthracis *from phenotypically similar *Bacillus *species isolates

**DOI:** 10.1186/1471-2180-6-22

**Published:** 2006-03-03

**Authors:** Chung K Marston, Jay E Gee, Tanja Popovic, Alex R Hoffmaster

**Affiliations:** 1 Meningitis and Special Pathogens Branch, Division of Bacterial and Mycotic Diseases, National Center for Infectious Diseases, Centers for Disease Control and Prevention, 1600 Clifton Rd., MS G34, Atlanta, Georgia, 30333, USA

## Abstract

**Background:**

*Bacillus anthracis *and *Bacillus cereus *can usually be distinguished by standard microbiological methods (e.g., motility, hemolysis, penicillin susceptibility and susceptibility to gamma phage) and PCR. However, we have identified 23 *Bacillus *spp. isolates that gave discrepant results when assayed by standard microbiological methods and PCR. We used multiple-locus variable-number tandem repeat analysis (MLVA), multiple-locus sequence typing (MLST), and phenotypic analysis to characterize these isolates, determine if they cluster phylogenetically and establish whether standard microbiological identification or PCR were associated with false positive/negative results.

**Results:**

Six isolates were LRN real-time PCR-positive but resistant to gamma phage; MLVA data supported the identification of these isolates as gamma phage-resistant *B. anthracis*. Seventeen isolates were LRN real-time PCR-negative but susceptible to gamma phage lysis; these isolates appear to be a group of unusual gamma phage-susceptible *B. cereus *isolates that are closely related to each other and to *B. anthracis*. All six *B. anthracis *MLVA chromosomal loci were amplified from one unusual gamma phage-susceptible, motile, *B. cereus *isolate (although the amplicons were atypical sizes), and when analyzed phylogenetically, clustered with *B. anthracis *by MLST.

**Conclusion:**

MLVA and MLST aided in the identification of these isolates when standard microbiological methods and PCR could not definitely identify or rule out *B. anthracis*. This study emphasized the need to perform multiple tests when attempting to identify *B. anthracis *since relying on a single assay remains problematic due to the diverse nature of bacteria.

## Background

*Bacillus anthracis *and *B. cereus *are two closely related gram-positive species belonging to the *B. cereus *group (*B. anthracis*, *B. cereus*, *B. thuringiensis*, *B. weihenstephanensis*, *B. pseudomycoides *and *B. mycoides*). Despite their close genetic relatedness, in general *B. anthracis *has several phenotypic characteristics making it easily differentiated from *B. cereus *and other members of the *B. cereus *group. *B. anthracis *is non-hemolytic on sheep blood agar, susceptible to penicillin, lysed by the gamma phage, and non-motile. Conversely, *B. cereus *is hemolytic on sheep blood agar, resistant to penicillin, resistant to lysis by the gamma phage, and motile. In addition, *B. anthracis *harbors two virulence plasmids, pXO1 and pXO2, which *B. cereus *lacks, although a recent reports have showed that rare *B. cereus *isolates harbor genes and similar plasmids closely related to pXO1 and pXO2 [1-6]. However, standard microbiologic identification can be complicated when isolates of *B. anthracis *are cured of both plasmids, are phage-resistant, or when rare non-hemolytic, gamma phage-susceptible *B. cereus *isolates are encountered [7].

Recently, more than 600 *B. anthracis *strains were recovered from a large collection of *Bacillus *spp. isolates that had been stored at the Centers for Disease Control and Prevention (CDC) from the 1950s through the 1980s [8]. All isolates were assayed using standard microbiological procedures, such as colony morphology on sheep blood agar (SBA) and the gamma phage susceptibility test [9]. In addition, all isolates were assayed using the *B. anthracis*-specific Laboratory Response Network (LRN) real-time PCR, which has been shown to have 100% sensitivity and specificity and includes three separate targets: one region on the *B. anthracis *chromosome and one region on each virulence plasmid, pXO1 and pXO2 [10]. During that study, 23 isolates were recovered that were not clearly identified or ruled out as *B. anthracis *due to discrepant results between standard microbiologic tests and the LRN PCR.

In the current study we used multiple-locus variable-number tandem repeat analysis (MLVA) and multiple-locus sequence typing (MLST) to identify and differentiate the 23 isolates that gave discrepant gamma phage susceptibility and PCR results. MLVA uses eight loci (six chromosomal and two plasmid) to discriminate *B. anthracis *isolates. MLVA has differentiated *B. anthracis *into 89 genotypes in two major clusters (A and B) and was used successfully to link clinical, powder, and environmental isolates from the 2001 anthrax outbreak [11,12]. In addition to its subtyping capabilities, Keim et al. reported that MLVA could be used to reliably identify *B. anthracis *because close relatives, such as *B. cereus *and *B. thuringiensis*, generally only amplify 0–2 MLVA loci, and any resulting allele sizes do not correspond to those observed in *B. anthracis *[12]. MLST is based on the sequencing of defined segments of seven housekeeping genes and also has recently been introduced as a typing method for members of the *B. cereus *group [13,14]. While this method is less discriminatory in differentiating *B. anthracis *isolates than MLVA, it has been used successfully to distinguish *B. anthracis *from its closest relatives within the *B. cereus *group. Both of these molecular methods, in addition to other standard microbiological methods, were used to characterize these 23 diagnostically challenging isolates, determine if they cluster phylogenetically, and establish whether standard microbiological identification or PCR were associated with false positive/negative results.

## Results

### Susceptibility to lysis by gamma phage, hemolysis, susceptibility to penicillin, and real-time PCR

The isolates in this study were chosen because they could not be definitively identified using gamma phage susceptibility and the LRN real-time PCR specific for *B. anthracis *which targets three genes: one on the *B. anthracis *chromosome and one on each virulence plasmid, pXO1 and pXO2. No isolates were positive for all three markers. Seventeen of the 23 isolates were susceptible to gamma phage lysis but gave negative results for all three LRN real-time PCR targets [Table 1]. Two of these isolates were beta-hemolytic on SBA, 14 were non-hemolytic, and one isolate was weakly hemolytic on SBA. The 14 non-hemolytic isolates had typical colony morphology of *B. anthracis *on an SBA plate, and with the exception of having negative PCR results, appeared to be consistent with *B. anthracis*. The remaining six isolates were resistant to gamma phage lysis and positive for the *B. anthracis *chromosomal marker used in the LRN real-time PCR. In addition to the chromosomal marker, two isolates were PCR-positive for the pXO2 marker and one isolate was positive for the pXO1 marker. The six gamma phage-resistant isolates were non-hemolytic and had the typical colony morphology for *B. anthracis *on SBA. All 23 isolates were susceptible to penicillin.

### MLVA

All 23 isolates were assayed by MLVA [Table 1]. All six PCR-positive, gamma phage-resistant isolates amplified all six VNTR loci located on the chromosome, indicating that these isolates were most likely gamma phage-resistant *B. anthracis*. In addition, one of the two plasmid markers was amplified in three of these isolates.

Among the 17 PCR-negative, gamma phage-susceptible isolates analyzed by MLVA, 16 isolates only amplified 0–1 of the eight VNTR loci. Thirteen isolates produced a *vrrA *282-bp amplicon, and one isolate produced a *vrrA *261-bp amplicon. However, neither of these sizes has been previously observed in *B. anthracis *strains [12]. Two isolates did not amplify any of the chromosomal VNTR loci and none of the 17 isolates amplified either plasmid VNTR loci. This data suggested that these isolates were gamma phage-susceptible *Bacillus *spp. and not *B. anthracis*. One PCR-negative, gamma phage-susceptible isolate, 2000031002, amplified all six chromosomal VNTR loci. However, four of the loci (CG3, *vrrB*_2_, *vrrB*_1_, and *vrrC*_1_) had previously unobserved allele sizes for *B. anthracis*. The other two loci were consistent with allele sizes previously seen in *B. anthracis*.

### MLST

**Figure 1:**
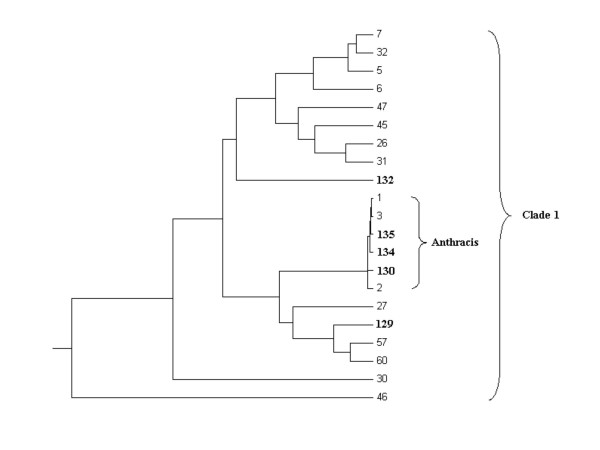
Dendrogram of UPGMA analysis in MEGA of concatenated sequences based on the Clade 1 STs in Priest et al.[8]. The five new STs determined in this study, ST-129, ST-130, ST-132, ST-134, and ST-135 are in bold.

All 23 isolates were assayed by MLST [Table 1]. The 17 PCR-negative, gamma phage-susceptible isolates were analyzed by MLST for further identification and to determine if these isolates were phylogenetically related. Three new sequence types (ST) were observed: ST 129, ST 130 and ST 132, all of which cluster in Clade 1, which includes the Anthracis lineage as defined by Priest et al. [Figure 1] [14]. Fifteen isolates were identified as ST 129. Of the 15 isolates, one was hemolytic; the remaining isolates were non-hemolytic. One weakly hemolytic isolate (2000031002) was designated ST 130, and an additional hemolytic isolate (2002013100) was designated ST 132. ST 129 and ST 132 clustered with other *B. cereus *STs in Clade 1, which includes the Anthracis lineage, but were sufficiently divergent from the *B. anthracis *STs, allowing clear differentiation. ST 130, however, grouped within the *B. anthracis *lineage [Figure 1] and is a double-locus variant of ST 1.

MLST performed on the six gamma phage-resistant isolates identified four isolates of ST 1 and single isolates of ST 134 and ST 135, both of which are new STs observed for *B. anthracis*. These three STs all cluster within the *B. anthracis *lineage [Figure 1].

## Discussion

Discrepancies between standard microbiologic identification and the LRN PCR results were observed for 23 isolates that were recovered after long-term storage. MLVA and MLST were performed to aid in resolving the discrepancies and identify these isolates. For the six gamma phage-resistant, PCR-positive isolates, all six chromosomal MLVA loci were successfully amplified supporting the identification of these isolates as gamma phage-resistant *B. anthracis*. Aside from being gamma phage-resistant, these isolates were typical *B. anthracis*. These isolates had the typical colony morphology, were non-hemolytic, susceptible to penicillin, and clustered with *B. anthracis *by MLST. All of the methods used in this study strongly support their identification as *B. anthracis*. Gamma phage-resistant *B. anthracis*, while uncommon, have been previously documented [15].

For 16 of the 17 gamma phage and penicillin susceptible, PCR-negative isolates, MLST analysis supports the identification of these isolates as *B. cereus *that are closely related to each other and to *B. anthracis *based on their STs and position in Clade 1 (as described by Priest et al.) [14]. While *B. cereus *and *B. thuringiensis *cannot be distinguished by MLST, Clade 1 is comprised of predominantly *B. anthracis *and *B. cereus *strains. The MLVA data also supported the identification of these isolates as *B. cereus*. Previous reports have documented the existence of gamma phage-susceptible *B. cereus *isolates such as these, but they are rarely encountered [7]. Since the identification of the *B. anthracis *gamma phage receptor has recently been characterized, it would be interesting to sequence the *gamR *gene of these isolates and to correlate these results with gamma phage susceptibility [16].

One PCR-negative, gamma phage-susceptible isolate, 2000031002, collected in 1960 from the Belgian Congo, provided the most peculiar results. This isolate was penicillin-susceptible, amplified all six MLVA chromosomal loci (although four allele sizes were unusual), and clustered with *B. anthracis *strains by MLST. It has been previously documented that a limited number of MLVA markers will amplify in *B. cereus *or *B. thuringiensis *[12], and a small number of closely related non-*B. anthracis *isolates have been identified that amplify all six chromosomal loci (although the alleles were sizes not previously seen in *B. anthracis*) [P. Keim, personal communication]. However, this isolate was weakly hemolytic and motile (data not shown). In addition, this isolate was PCR negative for both plasmids markers by the LRN real-time PCR and also did not amplify the MLVA plasmid markers. Although this isolate has several characteristics consistent with *B. anthracis*, based on the specificity of the PCR (as previously described and in this study), unusual VNTR allele sizes, and the lack of previous documentation of motile *B. anthracis *isolates, this isolate is most likely a very unusual *B. cereus*.

The isolates in this study are a small subset of a larger *Bacillus *spp. collection that was recovered after long-term storage (between 20–50 years) and limited descriptive information is available for many isolates in this collection [8]. For the isolates that we studied, we presume that the gamma phage-resistant *B. anthracis *were pathogenic when first isolated but lost the pXO1 and pXO2 plasmids during storage [8]. We presume that isolates of human and animal origin were fully virulent when first isolated as both plasmids are needed to cause disease. Marston et al. showed many of the isolates in the original collection had loss one or both virulence plasmids during storage [8]. In addition, environmental isolates were most likely virulent at the time of collection because these isolates followed the same pattern of plasmid loss over time as the clinical isolates in that study. For the *B. cereus *isolates, it is more difficult to ascertain their pathogenicity as *B. cereus *isolates can exhibit a wide range of virulence and cause a variety of infections [17]. Several methods suggest that isolates closely related to *B. anthracis *tend to be clinical (versus environmental) in nature [13,18,19]. However, MLST data has shown clinical isolates including isolates associated with severe disease are not clonal or restricted to the *B. anthracis *Clade[20]. Thus, pathogenicity can not be confidently deduced based on their phylogenetic relatedness.

In addition to having a negative result for the chromosomal marker, none of the gamma phage-susceptible *B. cereus *isolates were PCR positive for either plasmid marker. Their original identification was most likely based on phenotypic characteristics, as molecular methods such as PCR were not available at the time of collection and the plasmid profiles of these isolates could not be easily determined. Although *B. anthracis *and *B. cereus *are very closely related, they differ greatly in their pathogenicity which is generally attributed to the presence of plasmid encoded factors. For *B. anthracis*, these virulence factors are encoded on pXO1 and pXO2; however, recent reports have shown that other species can harbor genes and plasmids similar to pXO1 and pXO2 [1-6]. The possibility of horizontal transfer or curing of plasmids emphasizes the importance of using a chromosomal marker for the identification of *B. anthracis*.

## Conclusion

A primary concern when encountering unusual *Bacillus *isolates such as those included in this study is the need to positively identify or rule out *B. anthracis*. These isolates could not be definitively identified using gamma phage susceptibility, hemolysis on SBA, and penicillin susceptibility. *B*. *anthracis *isolates that are gamma phage-resistant or cured of both plasmids and rare non-hemolytic, gamma phage-susceptible *B. cereus *isolates can complicate the identification of *B*. *anthracis*. Rarely, *B. anthracis *can be resistant to gamma phage lysis (1% of *B. anthracis *isolates that were originally recovered from storage in our collection). In addition, some *B. cereus *isolates that are phylogenetically closely related to *B. anthracis *are gamma phage-susceptible. Interestingly, 15 of 17 gamma phage-susceptible isolates were indistinguishable by MLST (ST 129), and all three STs identified (ST 129, ST 130, and ST 132) cluster closely with *B. anthracis *in Clade 1. Thus, isolates that were closely related phylogenetically to *B. anthracis *were also phenotypically similar to *B. anthracis*. In this study, the combination of MLVA, MLST and the LRN PCR data were used to identify gamma phage-resistant *B. anthracis *isolates and *B. cereus *isolates phylogenetically and phenotypically very similar to *B. anthracis*. Although the PCR was found to be specific, our findings support our assertion that reliance on a single assay remains problematic due to the diverse nature of bacteria and concerns of potential bioengineering.

## Methods

### Bacterial isolates

From a collection of more than 600 isolates initially identified as *B. anthracis *at the time of isolation and storage, upon recovery, 23 isolates (4%) could not be definitively identified or ruled out as *B. anthracis *and were therefore further characterized in this study [Table 1]. With the exception of one isolate that was collected in 1974 (2002721544), all isolates were collected in the 1950s and 1960s. Source information was available for 10 isolates (9 environmental, 1 animal). The isolates were collected from five states throughout the U.S. and four different countries on three different continents.

### Susceptibility to lysis by gamma phage and hemolysis

One colony from each recovered isolate was inoculated heavily onto half of a sheep blood agar (SBA) plate. Subsequently, a single line perpendicular to the previously streaked area was streaked down the center of the plate. The second half of the plate was streaked to yield a confluent lawn, leaving approximately 1 cm between the two streaked halves. Each half of the SBA was inoculated with 5 μl of gamma phage. The plate then was marked to indicate where the phage was inoculated. The phage inoculum was allowed to dry, and the plate was incubated at 37°C for 18–24 h in ambient atmosphere. An isolate was considered susceptible to gamma phage lysis if there was a clear or partial plaque (area with no growth) on the SBA where the phage was inoculated. Gamma phage-resistant isolates had confluent growth at the point of gamma phage inoculation. Hemolysis of isolates was observed on the SBA and recorded after 18–24 h. For both assays, *B. anthracis *ATCC 4229 was used as a positive control, and *B. cereus *ATCC 14579 was used as a negative control.

### Real-time PCR

All isolates were assayed using the Laboratory Response Network (LRN) real-time PCR assay for *B. anthracis *as described by Hoffmaster et al., which targets three separate markers: the saspB gene on the *B. anthracis *chromosome, the pagA gene on pXO1, and the capA gene on pXO2 [10]. The assays were performed on the Lightcycler (Roche Diagnostics, Indianapolis, IN) or the Smart Cycler (Cepheid, Sunnyvale, CA) instruments. All three primer/probe sets (targeting both *B. anthracis *virulence plasmids and a chromosomal marker) were used.

### Susceptibility to penicillin

A confluent lawn of growth was streaked onto an SBA plate. One penicillin disk (10 U, Remel, Lenexa, KS) was placed on the agar. The SBA was incubated at 37°C for 24 h in ambient atmosphere. *B. cereus *ATCC 14579 and the *B. anthracis *ATCC 4229 were used as negative and positive controls, respectively. Isolates observed with any zones of inhibition were considered susceptible.

### Multiple-locus variable number tandem repeat analysis (MLVA)

MLVA was performed as previously described by Keim et al. [12]. Briefly, six chromosomal (*vrr*A, *vrr*B1, *vrr*B2, *vrr*C1, *vrr*C2, CG3) and two plasmid (pXO1-aat, pXO2-at) loci were amplified. Each amplicon was labeled with one of three dyes and analyzed on an ABI 377 automated DNA sequencer (Applied Biosystems, Foster City, CA). ABI Genescan software (Applied Biosystems) was used to analyze gel images.

### Multiple-locus sequence typing (MLST)

The MLST scheme described by Priest et al. was used in this study with the following modifications [14]. Seven housekeeping genes were sequenced: *glpF*, *gmk*, *ilvD*, *pta*, *pur*, *pycA*, *tpi*. Amplification was performed using a Roche Expand Hi Fidelity PCR System (Roche), with each reaction containing 200 μM dNTP mix, 0.4 μM of the forward and reverse primers, 1× reaction buffer (with MgCl_2_), 1.25 units of Expand Polymerase, and 1 μl of template [10]. PCR was performed on an ABI 2400 or 9700 thermalcycler (Applied Biosystems). Reactions were first incubated for 5 min at 95°C. Then 35 cycles were performed as follows: 15 sec at 94°C, 15 sec at the annealing temperatures noted in Priest et al. [14], except for *pta *which was annealed at 58°C, and extension for 1 min and 30 sec at 72°C. Reactions were then incubated at 72°C for an additional 5 min, with a final cooling to 4°C. PCR products were purified with a Qiaquick PCR purification kit (Qiagen, Valencia, CA).

Sequencing was performed using the ABI BigDye terminator cycle sequencing ver 3.1 kit per manufacturer's instructions, with the exception of using 3 μl of BigDye with 1× reaction buffer instead of 8 μl of BigDye alone (Applied Biosystems). Sequencing products were purified using Centri-Sep spin columns (Princeton Separations, Adelphia, NJ). These sequencing products were resolved using an ABI model 3100 automated DNA sequencing system (Applied Biosystems). Sequences were analyzed using the GCG Wisconsin package ver 10.1 (Accelrys, San Diego, CA), MEGA ver 2.1 [21], and by querying the *Bacillus *MLST database [22].

## Authors' contributions

CKM performed original recovery of isolates, standard microbiological procedures for identification of isolates, MLVA, data analysis and drafting of manuscript. JEG performed MLST, data analysis and drafting of manuscript. TP participated in the design and coordination of study and drafting of manuscript. ARH participated in conception of the study, its design, data analysis and drafting of the manuscript. This manuscript has been seen and approved by all authors.

## Figures and Tables

**Table 1:** Designation and characterization of 23 unusual *Bacillus *isolates recovered after long-term storage with conflicting PCR, gamma phage susceptibility, and colony morphology data.

**Strain designation**	**PCR results for chromosomal/pXO1/pXO2 markers**	**Hemolysis**	**Penicillin susceptibility**	**No. of chromosomal MLVA loci amplified**	**MLST ST**	**Final ID**
**Gamma phage-susceptible isolates**						

2000032803	(-)/(-)/(-)	Non-hemolytic	Susceptible	1	129	B. cereus*
2000032805	(-)/(-)/(-)	Non-hemolytic	Susceptible	1	129	B. cereus*
2000032847	(-)/(-)/(-)	Non-hemolytic	Susceptible	1	129	B. cereus*
2000032849	(-)/(-)/(-)	Non-hemolytic	Susceptible	1	129	B. cereus*
2000032884	(-)/(-)/(-)	Non-hemolytic	Susceptible	1	129	B. cereus*
2000032928	(-)/(-)/(-)	Non-hemolytic	Susceptible	1	129	B. cereus*
2000032947	(-)/(-)/(-)	Non-hemolytic	Susceptible	1	129	B. cereus*
2000032950	(-)/(-)/(-)	Non-hemolytic	Susceptible	1	129	B. cereus*
2002013138	(-)/(-)/(-)	Non-hemolytic	Susceptible	1	129	B. cereus*
2002013139	(-)/(-)/(-)	Non-hemolytic	Susceptible	1	129	B. cereus*
2002013140	(-)/(-)/(-)	Non-hemolytic	Susceptible	1	129	B. cereus*
2002013145	(-)/(-)/(-)	Non-hemolytic	Susceptible	1	129	B. cereus*
2002013146	(-)/(-)/(-)	Hemolytic	Susceptible	1	129	B. cereus*
2002013100	(-)/(-)/(-)	Hemolytic	Susceptible	1	132	B. cereus*
2000031002	(-)/(-)/(-)	Weakly hemolytic	Susceptible	6	130	B. cereus*
2000031061	(-)/(-)/(-)	Non-hemolytic	Susceptible	0	129	B. cereus*
2000031062	(-)/(-)/(-)	Non-hemolytic	Susceptible	0	129	B. cereus*
**Gamma phage-resistant isolates**						

2000031756	(+)/(-)/(-)	Non-hemolytic	Susceptible	6	1	*B. anthracis*
2000032841	(+)/(-)/(+)	Non-hemolytic	Susceptible	6	134	*B. anthracis*
2000032882	(+)/(-)/(+)	Non-hemolytic	Susceptible	6	1	*B. anthracis*
2002013066	(+)/(-)/(-)	Non-hemolytic	Susceptible	6	1	*B. anthracis*
2002721544	(+)/(+)/(-)	Non-hemolytic	Susceptible	6	1	*B. anthracis*
2002721585	(+)/(-)/(-)	Non-hemolytic	Susceptible	6	135	*B. anthracis*

*B. cereus and *B. thuringiensis *cannot be definitively differentiated by MLST or by other methods used in this study.

NA, not available
